# Characterization of guinea pig T cell responses elicited after EP-assisted delivery of DNA vaccines to the skin

**DOI:** 10.1016/j.vaccine.2016.11.052

**Published:** 2016-11-25

**Authors:** Katherine Schultheis, Hubert Schaefer, Bryan S. Yung, Janet Oh, Karuppiah Muthumani, Laurent Humeau, Kate E. Broderick, Trevor R.F. Smith

**Affiliations:** a Inovio Pharmaceuticals, Inc., 660W. Germantown Pike, Suite 110, Plymouth Meeting, PA 19462, USA; b Intracelluar Pathogens, Robert Koch Institute, Nordufer 20, 13353 Berlin, Germany; c Vaccine Center, The Wistar Institute of Anatomy & Biology, 3601 Spruce St., Philadelphia, PA 19104, USA

**Keywords:** Guinea pig, T cells, ELISpot, DNA vaccine, Electroporation, Skin

## Abstract

The skin is an ideal target tissue for vaccine delivery for a number of reasons. It is highly accessible, and most importantly, enriched in professional antigen presenting cells. Possessing strong similarities to human skin physiology and displaying a defined epidermis, the guinea pig is an appropriate model to study epidermal delivery of vaccine. However, whilst we have characterized the humoral responses in the guinea pig associated with skin vaccine protocols we have yet to investigate the T cell responses. In response to this inadequacy, we developed an IFN-γ ELISpot assay to characterize the cellular immune response in the peripheral blood of guinea pigs. Using a nucleoprotein (NP) influenza pDNA vaccination regimen, we characterized host T cell responses. After delivery of the DNA vaccine to the guinea pig epidermis we detected robust and rapid T cell responses. The levels of IFN-γ spot-forming units averaged approximately 5000 per million cells after two immunizations. These responses were broad in that multiple regions across the NP antigen elicited a T cell response. Interestingly, we identified a number of NP immunodominant T cell epitopes to be conserved across an outbred guinea pig population, a phenomenon which was also observed after immunization with a RSV DNA vaccine. We believe this data enhances our understanding of the cellular immune response elicited to a vaccine in guinea pigs, and globally, will advance the use of this model for vaccine development, especially those targeting skin as a delivery site.

## 1. Introduction

The skin is an attractive site for vaccination for several reasons, its accessibility lends itself to a less invasive and more tolerable vaccination site, the ability to directly monitor the site, and perhaps most importantly, the high number of resident professional antigen presenting cell (APC) populations at this site. Multiple pre-clinical experiments and clinical trials have demonstrated delivering a vaccine to the skin elicits robust immune responses in the host [1–7].

Historically the guinea pig (*Cavea porcellus*) has been one of the most widely used experimental animal models, so much that the term “guinea pig” has become a popular metaphor for scientific experimentation. The guinea pig model played an important role in the development of vaccines, including those targeting influenza, tuberculosis, diphtheria, and viral hemorrhagic fevers [8–11]. The guinea pig played an important role in the development of the two most widely used vaccines that are delivered at the skin, the BCG vaccine targeting tuberculosis and the rabies vaccine [12,13]. Unlike other small animal models such as the mouse, the guinea pig's skin possesses a defined epidermis, and is considered an optimal surrogate small animal model in terms of tissue physiology for preclinical vaccine studies targeting the epidermis. Furthermore the Hartley guinea pig strain is outbred, endowing further relevance on this animal as pre-clinical surrogate model for vaccine development.

We are currently developing a skin surface electroporation (SEP)-based platform to deliver DNA vaccines, and we have previously demonstrated the elicitation of robust humoral responses in guinea pigs after employing this delivery platform [2,14]. However, a limited catalogue of reagents available has hampered our ability to characterize vaccine-induced T cell responses in this model. Although a number of studies aimed at characterizing T cell responses in this important animal model have been reported [15–19], little is known concerning the cellular immune responses associated with skin vaccination in the guinea pig.

Here we describe the development of an IFN-γ ELISpot assay to quantify and monitor the cellular responses in a Hartley guinea pig model. Specifically, we evaluate cellular responses during a vaccination regimen with a pDNA vaccine encoding the Influenza nucle-oprotein (PR8) delivered to the skin of guinea pigs with the SEP device. Importantly, this assay uses peripheral blood cells so the kinetics of the host immune response elicited in a single guinea pig can be monitored by blood collection rather than sacrifice of multiple animals to remove lymphoid organs as a source of responder cells. We utilized this assay to characterize T cell responses elicited following pDNA vaccination. We proceeded to identify immunodominant T cell epitopes associated with responses against Influenza and RSV antigens in the animals immunized. Interestingly, in an outbred population of guinea pigs, all vaccinated animals displayed T cell responses against these epitopes. Data gathered in this study greatly increases our understanding of the cellular immune responses elicited by vaccination in a physiological relevant pre-clinical small animal model, and will greatly assist in expediting the translation of candidate vaccines into the clinic.

## 2. Materials and methods

### 2.1. Electroporation devices

The epidermal targeting surface EP (SEP) was an electrode array consisting of an array of gold-plated trocar needle of 0.43 mm diameter at a 1.5 mm spacing (Inovio Pharmaceuticals, Plymouth Meeting, PA). The SEP array is pressed down on the skin bleb made by Mantoux delivery of 50 μl plasmid formulation, in a manner in which all electrodes across the array contact the skin. The electrodes do not penetrate the live skin layers. Three individual 100 ms pulses of 25 V were delivered.

### 2.2. Animals

Female Hartley guinea pigs (8–10 weeks old) were purchased from Charles River Laboratories (Wilmington, MA). Animals were group housed with *ad libitum* access to food and water. Guinea pigs were group housed (4 per cage) and handled at BTS Research (San Diego, CA) according to the standards of the Institutional Animal Care and Use Committee (IACUC).

### 2.3. Plasmid DNA

NP vaccine plasmid encodes the full-length nucleoprotein derived from the A/Puerto Rico/8 (H1N1) strain of influenza. RSV-F vaccine plasmid contained an insert which was a consensus sequence of the RSV fusion glycoprotein of subtype A and B viruses. Sequences for the consensus strategy were obtained from GenBank. Consensus RSV-F was synthetically codon and RNA-optimized and then subcloned into a modified pVAX1 mammalian expression vector. All plasmids were diluted in 1×PBS before injection. In immunization studies 30 μg of pNP and 100 μg of pRSV-F was delivered.

### 2.4. Overlapping peptide pools

The influenza A virus (A/Puerto Rico/8/34(H1N1)) nucleocapsid protein peptide pools were created by synthesizing 120 individual 15mer peptides spanning the 498 amino acid sequence of the antigen. Peptides overlapped by 11 aa creating a 4 aa shift between next peptide in the sequence. Peptides were split into three pools each containing 40 individual peptides. The RSV-F peptide pool was matched to the consensus sequence of the RSV fusion glycoprotein of subtype A and B viruses. Peptides overlapped by 11 aa creating a 4 aa shift between next peptide in the sequence. Peptides were split into three pools each containing 20 individual peptides.

### 2.5. Endpoint-binding titer ELISA

Antibody responses against influenza NP and H5HA were performed as previously described [20]. Optical densities (OD) were read at 450 nm, and determined to be a positive titer if OD was two times that of background control. The bottom positive titer on the plate was plotted as the end-point titer.

### 2.6. Guinea pig IFN-γ ELISPOT assay

For the Interferon gamma ELISPOT with splenocytes, guinea pigs were euthanized and the spleens were harvested. Spleens were placed in 10 ml of cold PBS with 10% fetal bovine serum (FBS) and 1% (v/v) Penicillin/Streptomycin (R10 medium). Spleens were split in half, pummeled and passed through a 70 **u**m cell strainer to achieve single cell suspensions. For the IFN-γ ELISPOT with PBMCs three milliliter peripheral blood was drawn from the jugular vein of each anaesthetized animal and transferred immediately into EDTA blood collection tubes. Blood was diluted 1:1 with HBSS. Diluted Blood was layered over Ficoll-Paque Plus (GE Healthcare Life Sciences) and centrifuged (2000 rpm, 30 min, 24 °C). PBMCs were resuspended at 1×10^6^ cells/ml in R10 medium and plated at 100 μl/well on 96-well Millipore IP plates (Millipore) previously coated with 5 μg/ml primary anti-IFN-γ antibody V-E4 (antibodies for this assay were kindly provided by Dr. Schafer, Robert Koch Institute, Berlin, Germany) blocked with 10% (w/v) Sucrose and 2% (w/v) BSA (Sigma) in PBS. 100 μl of peptide or ConA stimulants were added to the cells. Samples were assayed in triplicates. After incubation in humidified 5% CO_2_ at 37 **°**C for 18 h, cells were removed by washing and 100 μl per well of 2 μg/ml biotinylated secondary anti-IFN-γ antibody N-G3 diluted in blocking buffer was added. Following a 2 h incubation and washing, alkaline phosphatase-conjugated streptavidin (R&D Systems Inc.) was added at 100 μl per well for 1 h at room temperature. Following washes, wells were incubated for 20 min at room temperature with 100 μl per well of BCIP/NBT detection reagent substrate (R&D Systems Inc.). Interferon-gamma positive spots were imaged, analyzed and counted using a CTL-Immunospot S6 ELISPOT Plate Reader and CTL-Immunospot software.

## 3. Results

### 3.1. Detection of cellular immune responses by IFN-γ ELISpot following pDNA immunization of guinea pigs

**Fig 1:**
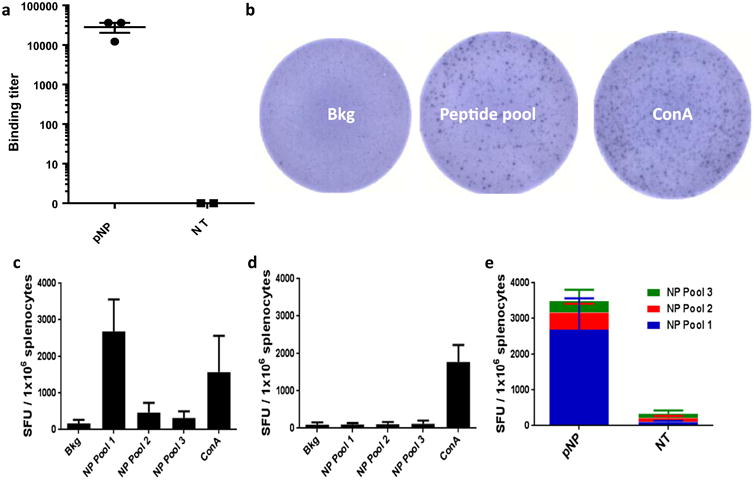
Development of an IFN-c ELISpot to detect cellular immune responses after pDNA immunization of guinea pigs. Hartley guinea pigs were immunized with 30 μg of a pDNA vaccine encoding the nucleoprotein of Influenza virus PR8 (pNP). Anti-Influenza PR8 nucleoprotein binding titers were detected in the serum of pNP immunized guinea pigs by ELISA (a). Splenocytes were stimulated overnight with peptide pools 1–3 spanning the NP antigen in wells coated with guinea pig anti-IFN-γ capture antibody. Spots forming units were detected after peptide and Con A stimulation using splenocytes isolated from a guinea pig immunized with pNP (b). Enumeration of IFN-γ spot forming units (SFU) for a pNP immunized (c) and non-treated (d) guinea pig. The average SFU's for each pool in the pNP immunized group versus the non-treated group is displayed (e). Mean SFU's ±SD are plotted. Three guinea pigs in the pNP group and two in the non-treated group. Data representative of two independent experiments.

With the aim of detecting antigen-specific T cell responses in the guinea pigs immunized with a plasmid DNA construct encoding the influenza nucleoprotein from the PR8 strain (pNP), we tested a detection and capture antibody pair – raised in mice, and recognizing conformation-specific epitopes on guinea pig IFN-γ - in an ELISpot assay [15,21]. First, to confirm the guinea pigs had mounted immune responses against the influenza NP antigen following this treatment regimen we analyzed antibody binding titers. Fig. 1a demonstrates all the pNP-immunized guinea pigs harbored IgG antibodies reactive to NP antigen (mean endpoint binding titer of 1:28,350). To determine whether these animals harbored antigen-specific T cell responses we sacrificed the guinea pigs, harvested their spleens and single cell suspensions were prepared. Splenocytes from individual guinea pigs were added to ELI-Spot plates coated with the IFN-γ capture antibody, and stimulated overnight with peptide pools containing 15mer peptides (overlapping by 11 amino acids), and spanning the entire Influenza NP PR8 protein encoded by the pNP vaccine. The peptides were split into three pools (as described in methods section). Representative visual images of IFN-γ spot forming units (SFU's) in wells containing non-stimulated, NP peptide Pool 1- or ConA-stimulated spleno-cytes from a pNP-vaccinated guinea pig are shown in Fig. 1b. An example enumeration of the IFN-γ ELISpot response in splenocytes harvested from a vaccinated animal (Fig. 1c) and a non-vaccinated animal (Fig. 1d) is shown. The IFN-γ response to peptides in Pool 1 (group mean of 2690 SFU's/10^6^ cells) dominated over Pool 2 (group mean of 470 SFU's/10^6^ cells) and Pool 3 (group mean of 330 SFU's/10^6^ cells) across the vaccinated group (Fig. 1c and e). In the non-vaccinated group the response against the NP peptide pools was not significantly higher than background levels (Fig. 1d and e). The cumulative mean response against Pools 1–3 spanning the entire NP antigen was 3490 SFU's/10^6^ cells in the vaccinated group, compared to 240 SFU's/10^6^ cells in the non-vaccinated group (Fig. 1e).

### 3.2. The kinetics of the cellular immune response elicited in a vaccination regimen delivering pNP ID with surface EP

**Fig 2:**
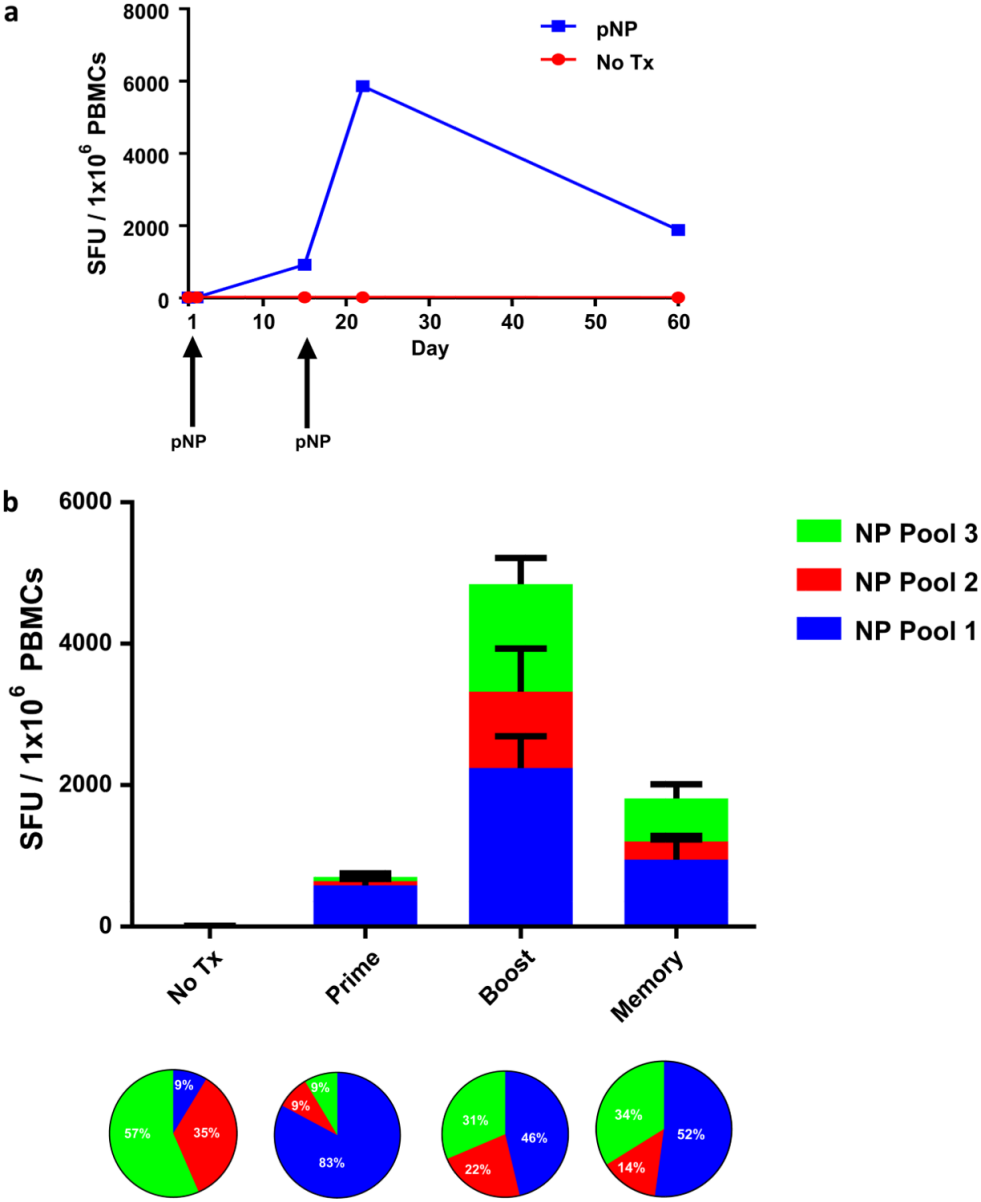
Robust IFN-c+ cellular immune responses are detected after delivery of pDNA vaccine to the skin with electroporation. On days 1 and 15 pNP DNA vaccine (30 lg) was delivered to the skin immediately followed by SEP electroporation. The PBMC IFN-γ ELISpot response was measured 14 days after the first immunization (prime), 7 days after the second (boost) and 46 days after the second (memory). (a) IFN-γ+ SFU's in an individual guinea pig. (b) Mean SFU's ±SEM are plotted for a group of 5 guinea pigs, along with pie charts indicating the percentage of the response generated by each pool.

The development of an effective vaccine product requires the ability to monitor both the immediate and long term levels of host immunity elicited following immunization. Ideally the investigator would be able to monitor the kinetics of the resulting immune response across a vaccination course in each individual animal. Sacrificing the animal to obtain the splenocytes will limit the analysis to a snapshot of the immune response mounted in an individual at a specific moment in time. To circumvent the need to sacrifice an animal to detect cellular immune responses, we collected whole blood samples from the jugular vein, and obtained PBMC populations by density gradient centrifugation. Collection of 3 ml of peripheral blood yielded between 2 and 4 million PBMCs. The antigen-specific IFN-γ ELISpot responses between splenocytes and PBMCs harvested from vaccinated animals were not significantly different (Supplemental Fig. 1). Thus, we proceeded with PBMCs harvested at defined time points to monitor the cellular immune responses elicited during a vaccination regimen. This regimen included the delivery of pNP vaccine to the abdominal skin of the guinea pig enhanced by a surface electroporation device (SEP). This EP device consists of a 4 **×** 4 array of electrodes, which makes direct contact with surface of skin where a bleb was made by the intradermal injection of 50 μl pDNA using the Mantoux technique [2,14]. The electrical field produced by activating the SEP device permits gene expression that is limited to the epidermis, and expression of the plasmid DNA is short-lived (approx. 7 days) due to the high turnover rate of cells in this tissue in the guinea pig [22]. The IFN-γ ELISpot responses measured during the vaccination regimen are presented in Fig. 2. Fig. 2a displays the IFN-γ cellular immune response kinetics to the NP peptide Pools 1-3 for an individual guinea pig. Fig. 2b displays the average response in a group of 5 guinea pigs. Robust IFN-γ + T cell responses (700 SFU's/10^6^ PBMCs) were detected 14 days after the first treatment.

On day 21, a strong boost response (4840 SFU's/10^6^ PBMCs) was observed seven days after the second immunization. The memory recall response detected at day 60 after the initial treatment was 1814 SFU's/10^6^ cells. The immune response against antigenic determinants in the influenza NP protein was broad. The response detected after the prime was focused upon peptide determinant/s in Pool 1 (83% of the total response), however the responses were more balanced after the boost (46% for Pool 1, 22% for Pool 2 and 31% for Pool 3) and memory time point (52% for Pool 1, 14% for Pool 2 and 34% for Pool 3). In summary, we detected strong and broad cellular immune responses to be elicited against influenza NP antigen after ID delivery of a pNP vaccine with SEP.

### 3.3. Influenza NP T cell epitope mapping

With the goal to further characterize the cellular immune response elicited in guinea pigs we aimed to identify MHC class I and MHC class II-presented epitopes recognized by CD8 and CD4 T cells, respectively. Due to the limits of blood draw (survival bleed not to exceed 10% of total circulating blood – as recommended by the Joint Working Group on Refinement, 1993) we could not harvest a sufficient number of PBMCs to analyze the responses to the total of 120 individual peptides in Pools 1, 2 and 3. We chose to analyze the IFN-γ responses to the individual peptides only in influenza NP peptide Pool 1 in pNP-vaccinated guinea pigs. Peptide Pool 1 was selected because approximately 50% of the total response (IFN-γ SFUs) to influenza NP PR8 antigen after pNP vaccination was to peptide determinant/s in this pool (Fig. 2). This suggested important T cell epitopes resided in this pool.

**Fig 3:**
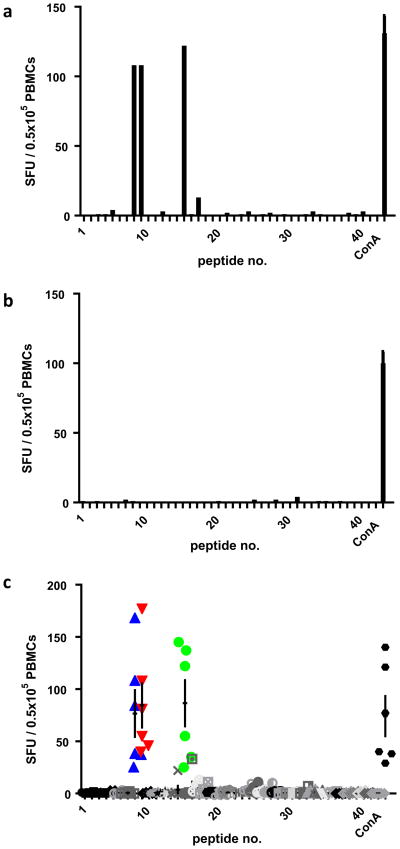
Antigenic peptide determinant mapping of the IFN-γ response after pNP immunization. PBMCs were harvested from non-treated or guinea pigs immunized with pNP, and plated into 42 wells. The PBMCs in wells 1–40 were stimulated as indicated with one of the individual 15mer peptides that span the influenza NP antigen Pool 1 region, cells in well 41 were not stimulated and well 42 were stimulated with ConA. (a) The enumeration of PBMC IFN-c SFU's recorded against each NP Pool 1 peptide from a pNP immunized, and (b) from a non-treated guinea pig is depicted. (c) IFN-γ SFU's from each pNP immunized guinea pig plotted. Mean SFU ±SEM against each peptide is displayed.

From animals which previously received two doses of the pNP vaccine delivered ID with SEP, PBMCs were harvested and stimulated with the 20 individual 15mer overlapping (4 aa shifts) pep-tides in Pool 1. These peptides spanned the first 175 aa of the NP PR8 antigen. Fig. 3a depicts the IFN-γ ELISpot response of an individual pNP-immunized guinea pig. Responses were observed against peptides 8 and 9 (2160 and 2160 SFU's/10^6^ cells, respectively), and peptide 15 (2440 SFU's/10^6^ cells). No responses were observed in untreated guinea pigs (Fig. 3b). In all pNP-immunized animals we observed responses against peptides 8 and 9, ranging between 500 and 3360 with a mean of 1533 SFU's/10^6^ cells for peptide 8, and ranging between 800 and 3540 with a mean of 1690 SFU's/10^6^ cells for peptide 9 (Fig. 3c). Additionally, all pNP-immunized animals responded to peptide 15, ranging between 500 and 2900 with a mean of 1730 SFU's/10^6^ cells (Fig. 3c).

**Fig 4:**
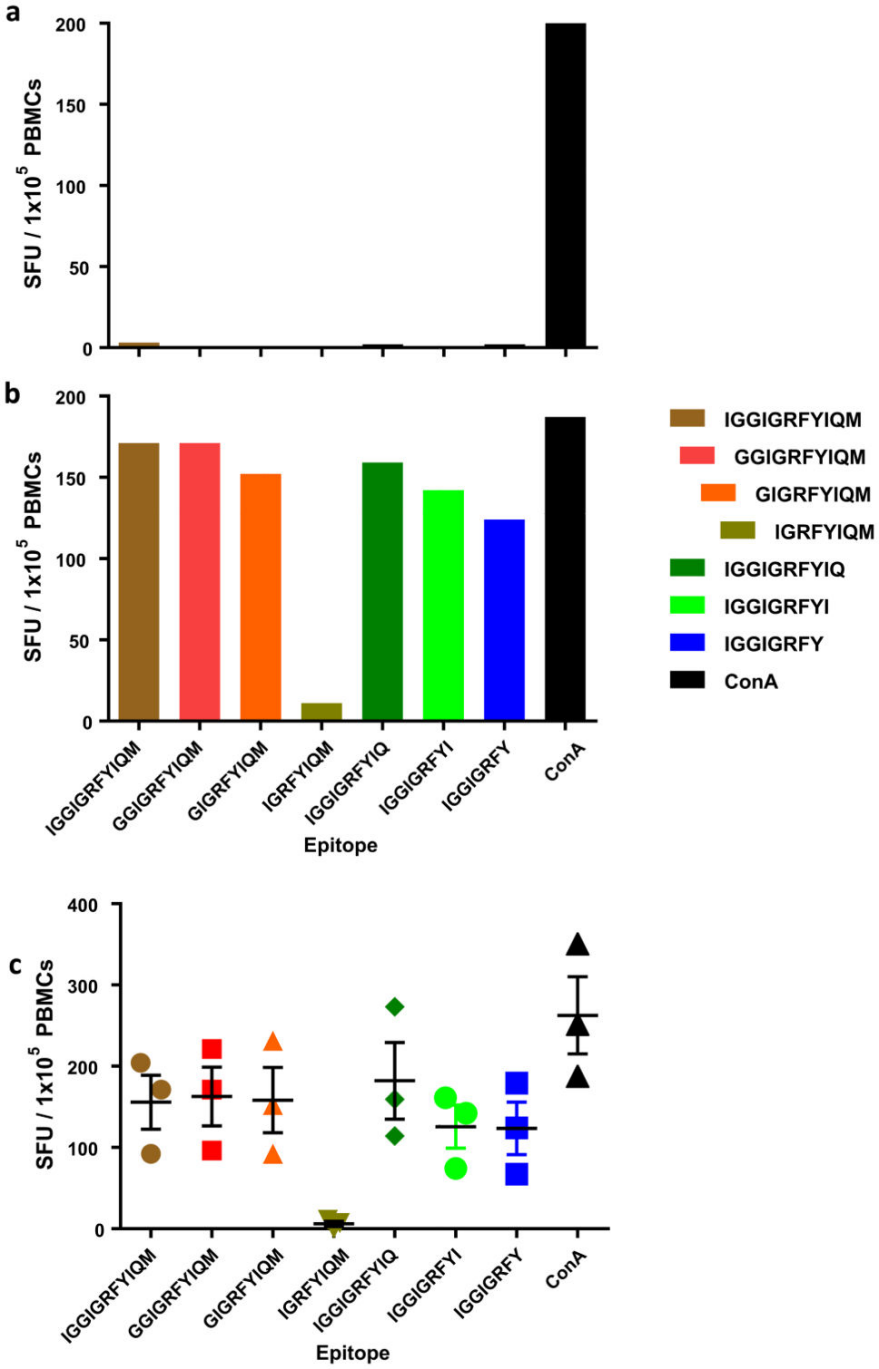
T cell epitope mapping of an immunodominant 6mer determinant after pNP immunization. The 11mer overlapping region shared between NP peptides 8 and 9 was truncated. In an IFN-γ ELISpot PBMCs from (a) non-treated and (b and c) pNP immunized guinea pigs were stimulated with the truncated peptide epitopes. IFN-γ SFU's were enumerated for a non-treated (a), a pNP immunized guinea pig (b). The responses from the three pNP immunized guinea pigs assayed are depicted in (c), with the mean SFU ±SD against each peptide displayed. Data indicated GIGRFY to be the immunodominant epitope.

Fig. 3 displays the levels of IFN-γ SFUs after stimulation with the adjacent peptides [8,9] to be equivalent (1533 and 1690 SFU's/10^6^, respectively), suggesting a shared epitope to be present in the overlapping region between the peptides. The length of this shared region was 11 aa. To identify this T cell epitope we truncated the 11mer from the N- and C-terminal, and stimulated PBMCs from a non-treated (Fig. 4a) and pNP-immunized (Fig. 4b) animal. All tested truncated versions of the IGGIGRFYIQM 11mer elicited IFN-γ SFUs of similar magnitude, except the 8mer IGRFYIQM which failed to elicit a detectable response (Fig. 4b). This finding was observed in all the pNP-immunized guinea pigs tested (Fig. 4c). Thus, peptide truncation analysis identified the 6mer GIGRFY to be an immunodominant epitope in the influenza NP PR8 antigen. The size of this epitope suggests a MHC class I determinant.

**Fig 5:**
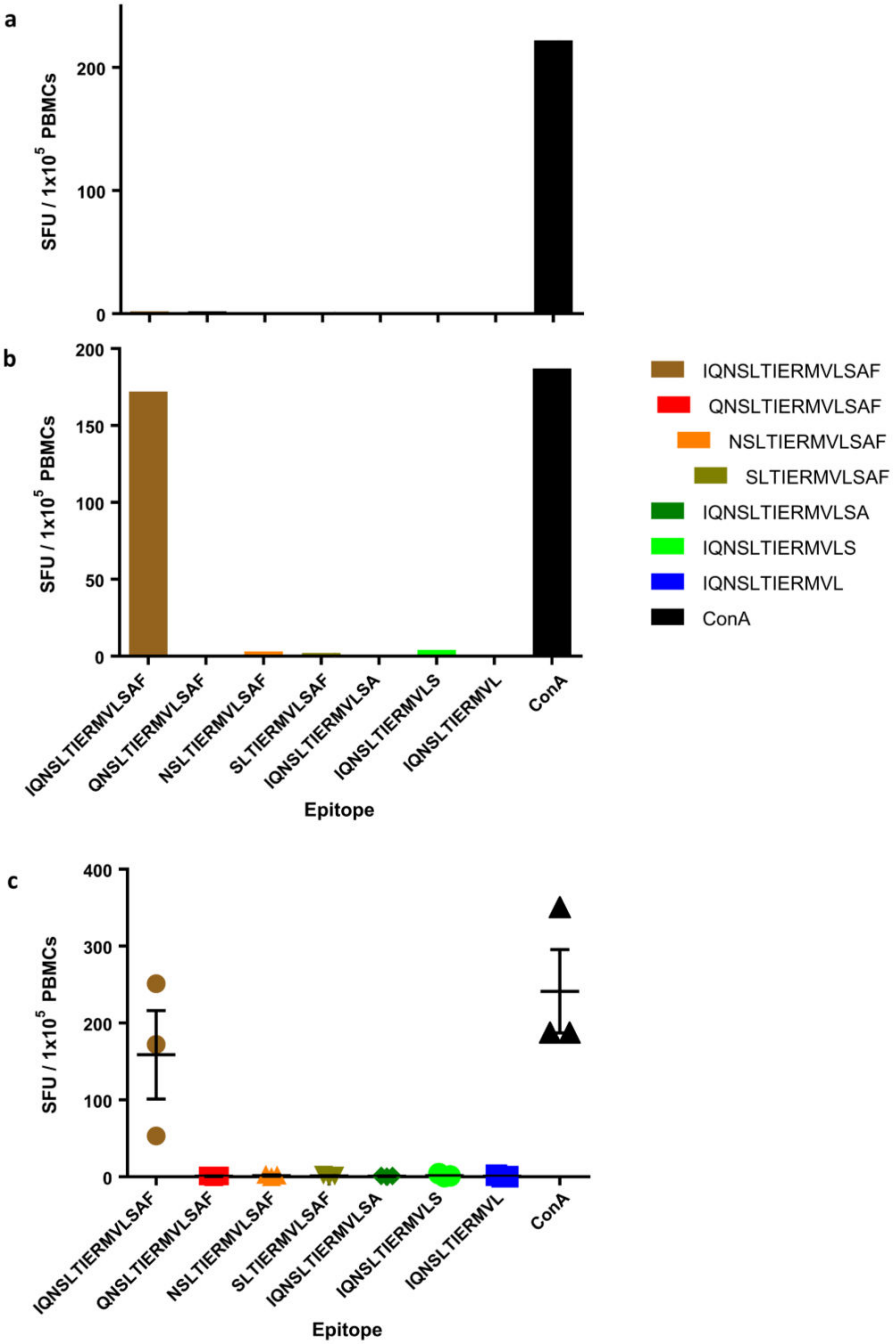
T cell epitope mapping of an immunodominant 15mer determinant after pNP immunization. Truncated amino acid sequences of the peptide number 15 in NP peptide Pool 1 were synthesized. In an IFN-γ ELISpot PBMCs from (a) non-treated and (b and c) pNP immunized guinea pigs were stimulated with the truncated peptide epitopes. IFN-γ SFU's were enumerated for a non-treated (a), a pNP immunized guinea pig (b). The responses from all three of the pNP guinea pigs are depicted in (c) with the mean SFU ±SD against each peptide displayed. Data indicated IQNSLTIERMVLSAF to be the immunodominant epitope.

Similar analysis of truncated versions of peptide 15 was performed to determine the associated T cell epitope (Fig. 5). No IFN-γ SFUs significantly above background were detected after stimulation of PBMCs form pNP-immunized animals with truncated versions of peptide 15 (Fig. 5a–c). Thus identifying the 15mer IQNSLTIERMVLSAF as the minimal determinant stimulating the IFN-γ response. MHC class II molecules generally present epi-topes between 12 and 16 aa's in length. This data strongly suggests this epitope to be a MHC II determinant recognized by CD4 + T cells.

### 3.4. Respiratory syncytial virus (RSV) F T cell epitope mapping

**Fig 6:**
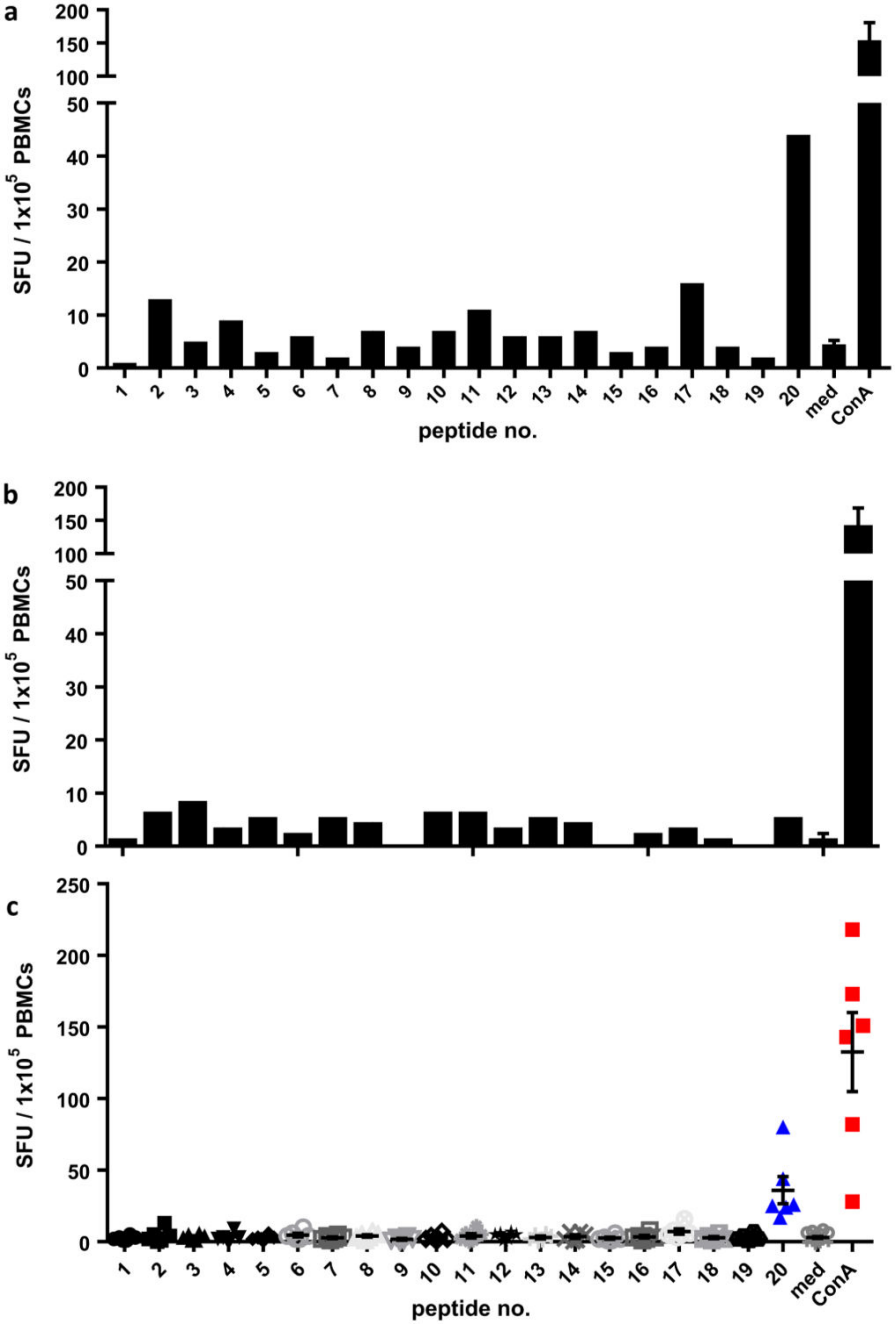
Identification of an immunodominant antigenic peptide determinant in guinea pigs immunized with pRSV-F. PBMCs were harvested from non-treated or guinea pigs after immunization with pRSV-F, and plated into 22 wells. The PBMCs in first 20 wells stimulated one of the individual 15mer peptides that span the RSV-F antigen Pool 1 region, cells in well 21 were not stimulated and in well 22 were stimulated with ConA. (a) The enumeration of PBMC IFN-γ SFU's recorded against each RSV-F Pool 1 peptide from a pRSV-F immunized, and (b) from a non-treated guinea pig is depicted. (c) IFN-γ SFU's the six pRSV-F immunized guinea pigs are plotted. Mean SFU ±SEM against each peptide is displayed.

Our findings with the influenza NP PR8 antigen suggested the same immunodominant epitopes within the virus-associated antigen may be targeted across the outbred Hartley guinea pig population. To test the generality of these findings to other respiratory viruses for which we lack an effective vaccine, we analyzed the IFN-c cellular immune responses to peptides within the respiratory syncytial virus (RSV) F protein after immunization with a pDNA vaccine encoding the RSV fusion protein (F). Responses against peptides in RSV F overlapping peptide library Pool 1 was chosen, as data suggested that determinant/s within this pool elicited the major portion of the detectable IFN-c ELISpot response in guinea pigs vaccinated with a plasmid encoding the RSV F antigen (see suppl. Fig. 2). Guinea pigs were treated with one dose of pRSV F delivered ID with SEP. Analysis of the IFN-c ELISpot responses against the individual 15mer overlapping peptides [1–20] which span Pool 1 of the RSV F antigen revealed an immun-odominant T cell epitope was residing within peptide number 20 (Fig. 6a and c). The response to this peptide in non-treated animals was not above background (Fig. 6b). All pRSV F-immunized animals responded to peptide 20, the mean response of the six guinea pigs was 360 SFU's/10^6^ cells, with a range of 170–800 SFU's/10^6^ cells (Fig. 6c). In summary, the results indicate the T cell responses driven by pDNA vaccines encoding respiratory disease virus associated antigens RSV F and influenza NP, are focused upon a limited number of immunodominant determinants in outbred Hartley guinea pigs.

## 4. Discussion

The induction of robust T cell responses has been described as essential to vaccine efficacy in many disease settings including, HIV, Influenza, Ebola and cancer [23]. As such the ability to assay and monitor these responses is critical to the vaccine development pathway. Here we describe a novel protocol for the assessment of cellular responses through an IFN-γ ELISPot in a guinea pig model. We believe this to be first example of a non-terminal approach to the assessment of vaccine-elicited T cell responses in guinea pigs. The guinea pig possesses skin of a similar thickness and structure to that of human skin, and is thus considered an optimal surrogate small animal model for vaccine delivery studies where the dermal compartment is the target for the vaccine delivery [24,25]. However, currently there is a paucity in tools to detect and characterize cellular immune responses in this animal. Here, to our knowledge for the first time, we report an IFN-γ ELISpot assay that can be used to detect T cell cellular responses to vaccine associated antigens in the peripheral blood. Specifically, we analyzed the kinetics of the cellular immune responses elicited following EP-enhanced pDNA delivery into the skin.

pDNA vaccination strategies offer significant advantages over the conventional attenuated or inactivated vaccines. pDNA vaccines have an excellent safety profile, can be manufactured to a large scale quickly, are easy to formulate, and can elicit both humoral and cellular responses. Importantly, DNA vaccines can be designed to express single or multiple target antigens of choice in a single formulation. However, the ability to efficiently deliver pDNA vaccines has been problematic, and was historically cited as the major reason for the low immune potency of this treatment in higher species [26]. As such, considerable effort has been attached to the development of enhanced delivery technologies to improve the uptake of pDNA in vivo in higher order animals. Delivery techniques, including electroporation [2,6,27,28], gene gun [29,30], tattooing [1,31] and microneedles [32], have been developed to reliably enhance gene expression in the skin tissue. The electroporation (EP) platform is a physical technique based on applying brief electrical pulses to the tissue of choice. Under the correct conditions this leads to cell membrane opening in a transient and reversible manner, facilitating the direct transport of pDNA into the cell. Upon comparison to naked DNA vaccination, a 10– 100 fold enhancement of the immunological response was observed when EP was employed as an enabling delivery technology [33–35]. While intramuscular (IM) EP has historically been the target tissue of choice, recently, considerable effort has been employed to develop intradermal (ID) EP techniques toward clinical applications [2,4,36,37]. The accessibility of the skin combined with the low penetration depths required for effective drug delivery, result in a less invasive and therefore potentially more tolerable clinical procedure [36,38]. The high number of resident professional antigen presenting cell populations endow the skin with very attractive characteristics for a target tissue for vaccine delivery. Multiple preclinical experiments and clinical trials have demonstrated that vaccinating in the skin elicits robust immune responses [1-7]. We have used both the intradermal invasive (CELLECTRA^®^-3P) and a surface (SEP) EP platforms to significantly enhance the expression of reporter gene plasmid in the skin and induce robust immunity [2,14,20,39,40]. While cellular responses in the dermally relevant non-human primate model can be easily assayed following immunization, the use of this model for vaccine screening protocols is limited due to the associated costs and availability of these species. The mouse is another model where the host cellular response is routinely monitored. However, the murine model lacks the relevant translation of skin physiology which limits its use for dermal vaccination protocols. Therefore, a greater insight of the cellular immune responses elicited following skin pDNA delivery enhanced by EP in a dermally clinically relevant small animal model, such as the guinea pig was needed. Here, we delineate for the first time the peripheral cellular immune responses driven by pDNA vaccination of the skin in the guinea pig. The ability to do this will greatly facilitate vaccine development studies based upon antigen delivery to the skin.

The results we gathered in this study concerning the magnitude of T cell responses in the guinea pig after skin vaccination were very encouraging. The magnitude of the T cell immune response to NP antigen was 4840 IFN-γ + SFU's/10^6^ PBMCs after second immunization. Furthermore, 14 days after one dose of pNP we observed levels averaging 700 IFN-c SFU's/10^6^ PBMCs, suggesting we were eliciting robust immune responses early in our immunization regimen. This observation is in line with previous skin vaccination studies by our group and others [1,14]. Our previous studies have demonstrated the specific delivery of pDNA into the epidermis permits the direct transfection of resident Langerhans cells. Further investigation delineated a mechanism via which motile guinea pig epidermal Langerhans cells expressing pDNA delivered by SEP, rapidly migrate out of the skin to the draining lymph nodes where they prime an adaptive immune response [14]. The ability to now monitor T cell and antibody responses in a more relevant pre-clinical surrogate model will significantly aide us in a more complete understanding of the character of the immune response elicited by our vaccine and how it correlates with disease protection. Future studies have been designed to determine whether delivery of pDNA vaccine with SEP provides early protection against influenza and Ebola virus challenge in pre-clinical models such as the guinea pig. Positive results will strengthen our case for an ID-delivered DNA vaccine as a suitable strategy to rapidly target emerging disease threats for example influenza pandemics, Ebola or MERS outbreaks. Importantly, protection of guinea pigs from Ebola challenge after skin vaccination with a DNA vaccine delivered with EP was recently reported [9]. Preliminary investigations of vaccination regimens in guinea pigs with Ebola DNA vaccines are showing very strong and rapid T cell responses (data not shown). Most importantly, we recently observed robust immune responses elicited to Ebola DNA vaccine delivered ID in humans (manuscript in preparation).

One very interesting observation we made in this study was the recognition of a limited number of conserved T cell epitopes across a group of outbred animals. These observations support the similar findings reported by Gillis et al. after vaccination of an outbred group of guinea pigs with CMV [15]. In our study, separate vaccination protocols delivering two different respiratory viral disease DNA vaccines, Influenza and RSV, suggested a number of immun-odominant epitopes were eliciting T cell responses. Epitope mapping analysis after pNP influenza immunization revealed two dominant epitopes to be present in the nucleoprotein 1–175 aa region of the NP PR8 antigen. The size of the identified epitopes strongly suggested a MHC class II epitope (IQNSLTIERMVLSAF) stimulating a CD4 + T cell response and a MHC class I epitope (GIGRFY) driving a CD8 + T cell response. However, we have not functionally confirmed these epitopes to be classical MHC class I or II-restricted. Thus we cannot rule out the possibility of these epitopes being associated with non-classical MHC molecules, such as class-Ib, which may present a less diverse repertoire of peptides and target more broad populations of CD8 + T cells [41]. Currently there is limited evidence that class Ib-restricted contribute to viral immunity. However, H2-M3-restricted CD8 + T cells have been induced after influenza virus stimulation [42,43], but their ability to kill virally infected cells has yet to be demonstrated. Furthermore, Hansen and colleagues recently reported broadly targeted CD8 + T cell responses restricted by MHC Ib molecule E in non-human primates immunized with a SIV-targeting vaccine [44]. Although it remains out of the scope of this study to further deter- mine the role of MHC in guinea pig T cell immunity, our observations and those by others should prompt further investigations [15].

In conclusion, we have described the use of a novel assay system to greatly enhance our ability detect and understand the cellular immune response in the very relevant, but underused, pre-clinical guinea pig model for skin vaccine development. We believe this study resulting in a working protocol will aide in the advancement of the guinea pig as tool for vaccine development. By utilizing the described IFN-γ ELISpot assay we have gained the ability to refine our skin vaccination research efforts in a highly relevant surrogate model without the need to sacrifice the animal. Finally, we have highlighted the ability of pDNA vaccines delivered to the epidermis of the skin, with a non-invasive EP device to drive high magnitude T cell responses.

## Supplementary Material

Supplemental files

## Abbreviations

EP: electroporation
NP: nucleoprotein
SEP: surface electropora-tion
SFU: spot forming unit
RSV: respiratory syncytial virus

## References

[1] Bins AD, Jorritsma A, Wolkers MC, Hung CF, Wu TC, Schumacher TN (2005). A rapid and potent DNA vaccination strategy defined by in vivo monitoring of antigen expression. Nat Med.

[2] Broderick KE, Shen X, Soderholm J, Lin F, McCoy J, Khan AS (2011). Prototype development and preclinical immunogenicity analysis of a novel minimally invasive electroporation device. Gene Ther.

[3] Song JM, Kim YC, Barlow PG, Hossain MJ, Park KM, Donis RO (2010). Improved protection against avian influenza H5N1 virus by a single vaccination with virus-like particles in skin using microneedles. Antiviral Res.

[4] Brave A, Nystrom S, Roos AK, Applequist SE (2011). Plasmid DNA vaccination using skin electroporation promotes poly-functional CD4 T-cell responses. Immunol Cell Biol.

[5] Bakari M, Aboud S, Nilsson C, Francis J, Buma D, Moshiro C (2011). Broad andpotent immune responses to a low dose intradermal HIV-1 DNA boosted withHIV-1 recombinant MVA among healthy adults in Tanzania. Vaccine.

[6] Drabick JJ, Glasspool-Malone J, King A, Malone RW (2001). Cutaneous transfection andimmune responses to intradermal nucleic acid vaccination are significantly enhanced by in vivo electropermeabilization. Mol Ther.

[7] Glenn GM, Taylor DN, Li X, Frankel S, Montemarano A, Alving CR (2000). Transcutaneous immunization: a human vaccine delivery strategy using a patch. Nat Med.

[8] Orme IM (2005). The use of animal models to guide rational vaccine design. Microbes Infect.

[9] Shedlock DJ, Aviles J, Talbott KT, Wong G, Wu SJ, Villarreal DO (2013). Induction of broad cytotoxic T cells by protective DNA vaccination against Marburg and Ebola. Mol Ther.

[10] Padilla-Carlin DJ, McMurray DN, Hickey AJ (2008). The guinea pig as a model of infectious diseases. Comp Med.

[11] Kong H, Zhang Q, Gu C, Shi J, Deng G, Ma S (2015). A live attenuated vaccine prevents replication and transmission of H7N9 virus in mammals. Sci Rep.

[12] Veeraraghavan N (1959). Improvement of the antigenicity of antirabies vaccine bypooling checked by post-challenge vaccination of guinea-pigs. Bull WorldHealth Organ.

[13] EXPERIMENTAL studies of vaccination, allergy, and immunity in tuberculosis. II. Effect of varying the dose of BCG. Bull World Health Organ.

[14] Smith TR, Schultheis K, Kiosses WB, Amante DH, Mendoza JM, Stone JC (2014). DNA vaccination strategy targets epidermal dendritic cells, initiating their migration and induction of a host immune response. Mol Ther Methods Clin Dev.

[15] Gillis PA, Hernandez-Alvarado N, Gnanandarajah JS, Wussow F, Diamond DJ, Schleiss MR (2014). Development of a novel, guinea pig-specific IFN-gamma ELISPOT assay and characterization of guinea pig cytomegalovirus GP83-specific cellular immune responses following immunization with a modified vaccinia virus Ankara (MVA)-vectored GP83 vaccine. Vaccine.

[16] Hayward AR, Burger R, Scheper R, Arvin AM (1991). Major histocompatibility complex restriction of T-cell responses to varicella-zoster virus in guinea pigs. J Virol.

[17] Grover A, Taylor J, Troudt J, Keyser A, Arnett K, Izzo L (2009). Kinetics of the immune response profile in guinea pigs after vaccination with *Mycobacteriumbovis* BCG and infection with Mycobacterium tuberculosis. Infect Immun.

[18] Klunner T, Bartels T, Vordermeier M, Burger R, Schafer H (2001). Immune reactions of CD4- and CD8-positive T cell subpopulations in spleen and lymph nodes of guinea pigs after vaccination with *Bacillus Calmette Guerin*. Vaccine.

[19] Schafer H, Scheper RJ, Borsdorf B, Burger R (2003). Effector functions of CD8-positiveguinea pig T lymphocytes. Cell Immunol.

[20] Lin F, Shen X, McCoy JR, Mendoza JM, Yan J, Kemmerrer SV (2011). A novelprototype device for electroporation-enhanced DNA vaccine deliverysimultaneously to both skin and muscle. Vaccine.

[21] Schafer H, Kliem G, Kropp B, Burger R (2007). Monoclonal antibodies to guinea piginterferon-gamma: tools for cytokine detection and neutralization. J Immunol Methods.

[22] Mendoza J, Amante D, Kichaev G, Knott C, Kiosses W, Smith T (2013). Elucidatingthe kinetics of expression and immune cell infiltration resulting from plasmidgene delivery enhanced by surface dermal electroporation. Vaccines.

[23] Gilbert SC (2012). T-cell-inducing vaccines - what's the future. Immunology.

[24] Barbero AM, Frasch HF (2009). Pig and guinea pig skin as surrogates for human in vitro penetration studies: a quantitative review. Toxicol In Vitro.

[25] Panchagnula R, Stemmer K, Ritschel WA (1997). Animal models for transdermal drugdelivery. Methods Find Exp Clin Pharmacol.

[26] Weiner DB (2008). DNA vaccines: crossing a line in the sand. Introduction to specialissue Vaccine.

[27] Guo S, Donate A, Basu G, Lundberg C, Heller L, Heller R (2011). Electro-gene transfer toskin using a noninvasive multielectrode array. J Control Release.

[28] Roos AK, Eriksson F, Timmons JA, Gerhardt J, Nyman U, Gudmundsdotter L (2009). Skin electroporation: effects on transgene expression, DNA persistence and local tissue environment. PLoS ONE.

[29] Klein RM, Wolf ED, Wu R, Sanford JC (1992). High-velocity microprojectiles fordelivering nucleic acids into living cells. Biotechnology.

[30] Yang NS, Burkholder J, Roberts B, Martinell B, McCabe D (1990). In vivo and in vitro gene transfer to mammalian somatic cells by particle bombardment. Proc Natl Acad Sci USA.

[31] Oosterhuis K, van den Berg JH, Schumacher TN, Haanen JB (2012). DNA vaccines andintradermal vaccination by DNA tattooing. Curr Top Microbiol Immunol.

[32] Prausnitz MR (2004). Microneedles for transdermal drug delivery. Adv Drug Deliv Rev.

[33] Sardesai NY, Weiner DB (2011). Electroporation delivery of DNA vaccines: prospectsfor success. Curr Opin Immunol.

[34] van Drunen Littel-van den Hurk S, Hannaman D (2010). Electroporation for DNAimmunization: clinical application. Expert Rev Vaccines.

[35] Hirao LA, Wu L, Satishchandran A, Khan AS, Draghia-Akli R, Finnefrock AC (2010). Comparative analysis of immune responses induced by vaccination with SIV antigens by recombinant Ad5 vector or plasmid DNA in rhesus macaques. Mol Ther.

[36] Diehl MC, Lee JC, Daniels SE, Tebas P, Khan AS, Giffear M (2013). Tolerability ofintramuscular and intradermal delivery by CELLECTRA adaptive constantcurrent electroporation device in healthy volunteers. Hum Vaccin Immunother.

[37] El-Kamary SS, Billington M, Deitz S, Colby E, Rhinehart H, Wu Y (2012). Safety and tolerability of the Easy Vax clinical epidermal electroporation system in healthy adults. Mol Ther.

[38] Kim YC, Jarrahian C, Zehrung D, Mitragotri S, Prausnitz MR (2012). Delivery systems for intradermal vaccination. Curr Top Microbiol Immunol.

[39] Donate A, Coppola D, Cruz Y, Heller R (2011). Evaluation of a novel non-penetratingelectrode for use in DNA vaccination. PLoS ONE.

[40] Lin F, Shen X, Kichaev G, Mendoza JM, Yang M, Armendi P (2012). Optimizationof electroporation-enhanced intradermal delivery of DNA vaccine using a minimally invasive surface device. Hum Gene Ther Methods.

[41] Rodgers JR, Cook RG (2005). MHC class Ib molecules bridge innate and acquired immunity. Nat Rev Immunol.

[42] Byers DE, Fischer Lindahl K (1998). H2–M3 presents a nonformylated viral epitope toCTLs generated in vitro. J Immunol.

[43] Milligan GN, Flaherty L, Braciale VL, Braciale TJ (1991). Nonconventional (TL-encoded) major histocompatibility complex molecules present processed viral antigen to cytotoxic T lymphocytes. J Exp Med.

[44] Hansen SG, Wu HL, Burwitz BJ, Hughes CM, Hammond KB, Ventura AB (2016). Broadly targeted CD8^+^ T cell responses restricted by major histocompatibility complex E. Science.

