# Supplementary material for: Characterization of guinea pig T cell responses elicited after EP-assisted delivery of DNA vaccines to the skin
Source: Vaccine. Author manuscript; Available in PMC 2017 Jan 9. (PMC5221502; doi:10.1016/j.vaccine.2016.11.052)
Supplement: Supplemental files [file NIHMS838001-supplement-Supplemental_files.pdf]

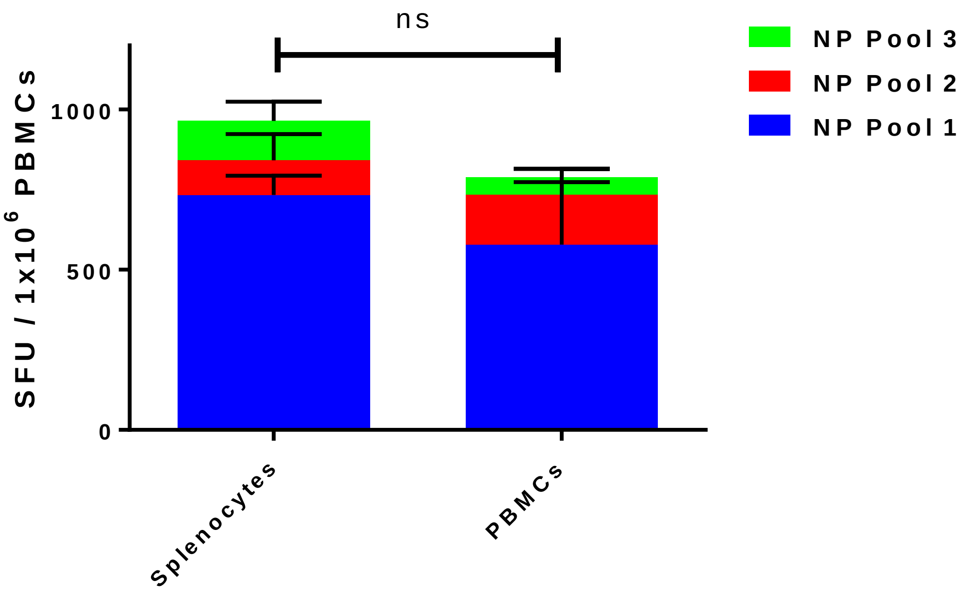

Supplemental Figure 1.

IFN-γ+ ELISpot responses in the splenocyte and PBMC populations of pNP-immunized Guinea Pigs.

Splenocytes and PBMCs were harvested from Guinea pigs after immunization with pNP, and stimulated with Influenza NP antigen peptide Pools 1 to 3. Mean (+/-SEM) IFN-γ SFU's for a group of 5 pNP-immunized guinea pigs are plotted.

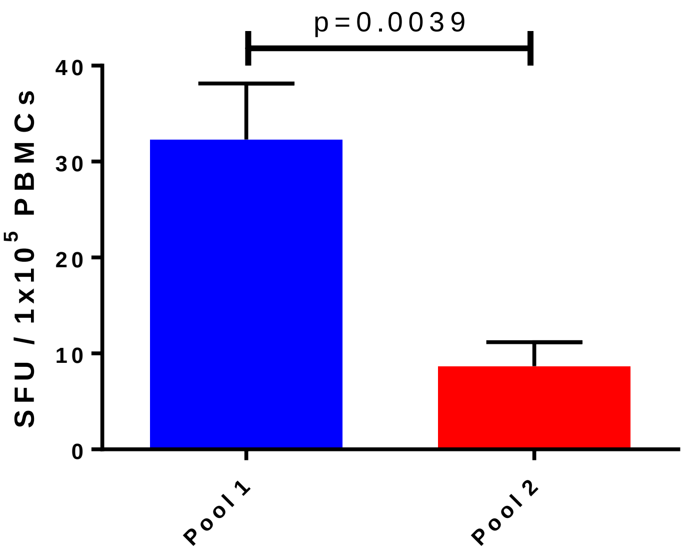

Supplemental Figure 2.

PBMC IFN-γ+ ELISpot responses to RSV-F antigen peptide Pools 1&2.

PBMCs were harvested from Guinea pigs after immunization with pRSV-F, and stimulated with RSV-F antigen peptide Pool 1 or 2. Mean (+/-SEM) IFN-γ SFU's for a group of 5 pRSV-F immunized guinea pigs are plotted.
